# Association of the TRIM family protein with survival outcomes and clinicopathological features in colorectal cancer: a systematic review and meta-analysis

**DOI:** 10.1186/s12885-024-12280-z

**Published:** 2024-04-27

**Authors:** Ying Wu, Chen Chen, Xian Hua, Chunhua Zhao, Han Min

**Affiliations:** 1 Department of Gastroenterology, The Affiliated Suzhou Hospital of Nanjing Medical University, Suzhou Municipal Hospital, Gusu School, Nanjing Medical University, Suzhou, Jiangsu China; 2 Department of General Medicine, Big Data Center, The Affiliated Suzhou Hospital of Nanjing Medical University, Suzhou Municipal Hospital, Nanjing Medical University, Suzhou, China

**Keywords:** Colorectal cancer, TRIM family proteins, Prognosis, Clinicopathological features

## Abstract

**Background:**

The tripartite motif (TRIM) proteins have been reported to play crucial roles in various malignancies. However, the clinical significance of TRIM proteins in colorectal cancer (CRC) remains controversial. This study aimed to evaluate the association between TRIM proteins and the clinicopathological features and survival outcomes in patients with CRC.

**Methods:**

We performed a meta-analysis to investigate whether TRIM is a prognostic factor in CRC. PubMed, Embase, Web of Science, CNKI and Weipu databases were searched to identify eligible studies that evaluated the association between TRIM proteins and overall survival (OS), as well as the clinicopathological features of patients with CRC. Hazard ratios (HR) or odds ratios (OR) with 95% confidence interval (CI) were derived and pooled using a fixed-effects model.

**Results:**

From inception to March 2023, we extracted study characteristics and prognostic data for each identified study. Twelve studies enrolling 1608 patients were eligible for inclusion. Data on OS and recurrence-free survival (RFS) were available for 12 and 2 studies, respectively. The pooled analysis results showed a significant correlation between the elevated TRIM proteins and shorter OS (HR = 2.42, 95% CI: 1.96–2.99) and worse RFS (HR = 2.51, 95% CI: 1.78–3.54) in patients with CRC. The combined ORs indicated that TRIM protein over-expression was significantly associated with advanced TNM stage (OR = 2.26, 95% CI: 1.25–4.10), deep tumor invasion (OR = 2.01, 95% CI: 1.04–3.88), lymph node metastasis (OR = 2.99, 95% CI: 2.19–4.09) and perineural invasion (OR = 1.95, 95% CI: 1.18–3.23).

**Conclusions:**

Our findings suggest that TRIM proteins can predict tumor progression and poor prognosis in CRC. Therefore, TRIM proteins may be promising therapeutic targets for patients with CRC.

**Graphical Abstract:**

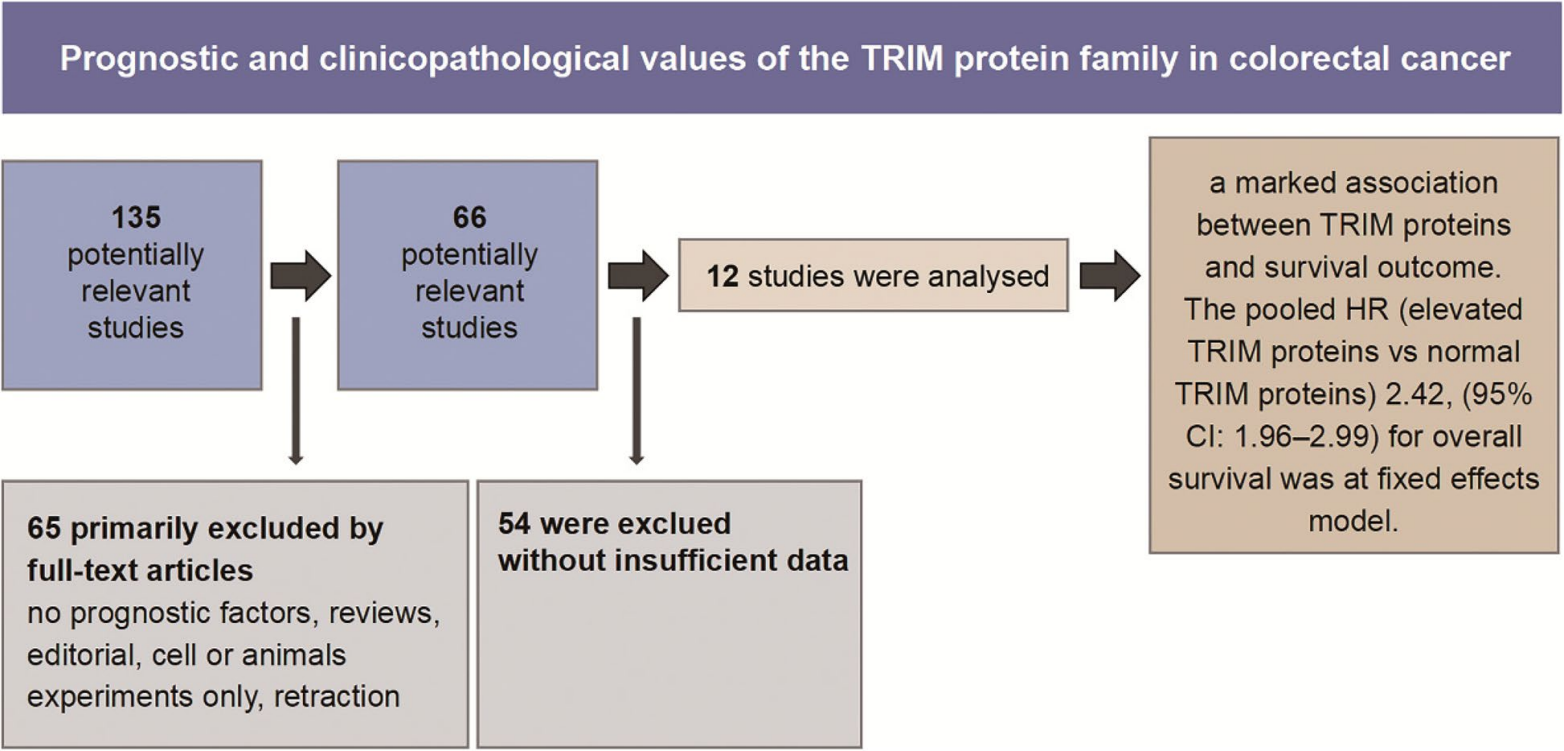

**Supplementary Information:**

The online version contains supplementary material available at 10.1186/s12885-024-12280-z.

## Introduction

Colorectal cancer (CRC) is a common malignant cancer in both males and females and is the second leading cause of cancer-related deaths worldwide [1]. Despite a slight decrease in mortality due to enhanced detection techniques and ever-improving therapeutic approaches, patients with CRC still exhibit poor survival owing to the rising metastatic capacity of primary tumors and high recurrence rate [2, 3]. Recently, numerous studies have concentrated on novel biomarkers for the clinicopathological characteristics and prognosis of patients with CRC, with the aim of identifying new therapeutic targets [4, 5].

The tripartite motif (TRIM) family of proteins is characterized by an N-terminal TRIM containing a RING-finger domain, one or two zinc-finger domains known as a B-box and a coiled-coil region [6]. Owing to the RING-finger domain, TRIM proteins possess E3 ligase activity and participate in the degradation of a series of proteins. TRIM proteins have been reported to play crucial roles in various biological behaviors, and their dysregulation contributes to oncogenesis and tumor progression [7, 8]. Several studies have examined the prognostic role of different TRIM proteins in various malignancies [9–11]. However, the prognostic role of the TRIM protein family in CRC remains unclear.

Therefore, we conducted a systematic review and meta-analysis to evaluate the association between TRIM proteins and the clinical outcomes in patients with CRC. Understanding TRIM proteins can considerably contribute to their validations as promising novel biomarkers and potential therapeutic targets for CRC.

## Materials and methods

The present study was conducted according to the Preferred Reporting Items for Systematic Reviews and Meta-Analyses [12]. We registered on PROSPERO (CRD42023417799) before conducting the literature search.

### Search strategy

Two independent researchers systematically searched the Web of Science, PubMed, Embase, CNKI and Weipu electronic databases from their inception to March 1, 2023. No language restrictions were imposed. The following medical subject headings or keywords were adopted according to the retrieval strategy: ‘trim’ OR ‘trim family’ OR ‘trim proteins’ OR ‘tripartite motif’ OR ‘tripartite motif proteins’; ‘cancer’ OR ‘tumor’ OR ‘carcinoma’ OR ‘neoplasm’; ‘colon’ OR ‘rectum’ OR ‘colorectal’. To avoid ignoring qualified studies, all references of the selected primary studies were screened.

### Inclusion and exclusion criteria

First, based on the pre-specified selection criteria, two independent researchers screened titles and abstracts to exclude irrelevant studies. Duplicate studies were also excluded from analysis. Next, the full text of all potentially pertinent studies were assessed according to the following inclusion criteria: (1) the expression of TRIM proteins was detected in primary tumor tissues after surgical resection; (2) all patients included were divided into two or more groups based on the TRIM expression levels; (3) the hazard ratio (HR) of survival outcomes or clinicopathological features related to high and low levels of TRIM expressions were available; and (4) the survival curves or sufficient data were available to calculate the HR with 95% confidence interval (CI).

The exclusion criteria were as follows: (1) studies without prognostic outcomes; (2) studies with insufficient data for analysis; and (3) study designs of reviews, case reports, conference abstracts, editorials and letters.

### Data extraction

The following data were independently collected by two investigators from eligible studies: first author, year of publication, country, sample capacity, duration of follow-up, overexpression rate, detection method and outcome measures. The data were extracted into a predefined table. Additionally, information was collected on the clinicopathological parameters related to tumor progression. For survival outcome extraction, the HRs and 95% CIs were directly collected from the multivariate and univariate analysis calculated by Cox proportional hazards regression model. We extracted the crude HR and 95% CIs from the univariate analysis and the adjusted HR and 95% CIs after adjustment for potential confounders from the multivariate analysis. Engauge Digitizer version 4.0. was used to estimate survival statistics if Kaplan–Meier curves were only provided. Disagreements between the two reviewers were verified after a discussion and review of the trial information.

### Quality assessment

The quality of the enrolled studies was assessed using the Newcastle–Ottawa quality assessment scale, which consists of three main aspects: (1) selection of the research groups, (2) comparability of the groups and (3) ascertainment of the exposure or outcome [13].

### Statistical analysis

The relationship between the expression of TRIM proteins and prognosis, as well as the clinicopathological features of CRCs, was analyzed using STATA/SE 17. To estimate the potential heterogeneity among the included studies, I^2^ statistics and the Chi-square Q test were employed. A fixed-effects model was applied to pool the HR of each eligible study into a summary HR if there was no obvious heterogeneity (I^2^ < 50% or *P*_Q_ ≥ 0.10). In contrast, when heterogeneity was considered significant (I^2^ ≥ 50% or *P*_Q_ < 0.10), the random-effects model was adopted. The sources of heterogeneity were explored using subgroup or meta-regression analyses to examine all possible factors. Funnel plots and Egger’s and Begg’s tests were performed to assess the risk of publication bias [14]. Sensitivity analysis was performed to ensure the stability of the summarized results. In the present study, *P < 0.05 indicated a significant difference.

## Results

### Characteristics of eligible studies

**Fig. 1:**
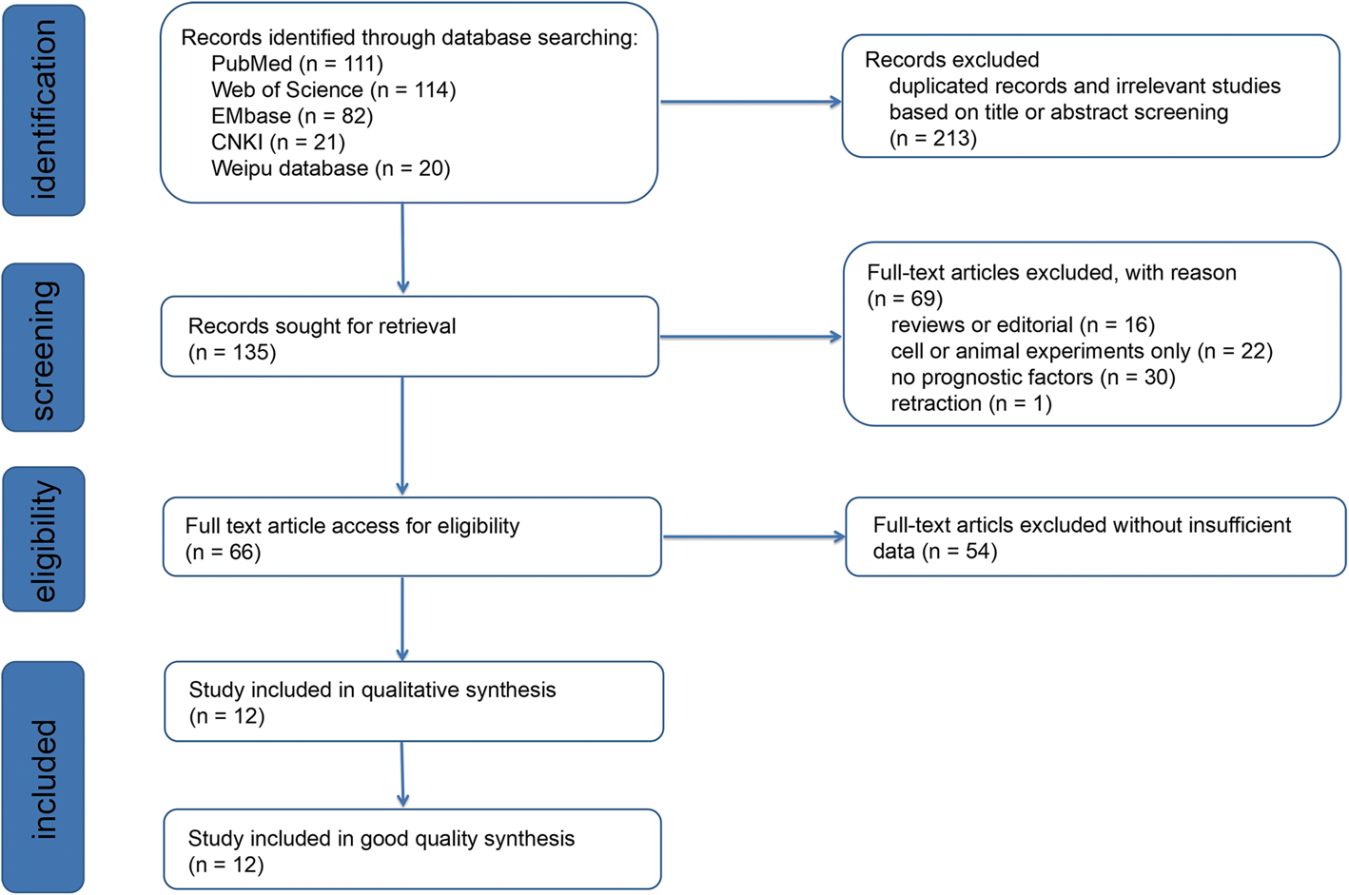
Flow-process diagram of the study selection process

Details of the meta-analysis search process are shown in Fig. 1. Using a database search, 135 articles were initially identified, after removing duplicates. Following the removal of reviews and articles focusing on cellular or animal experiments, 66 studies remained for further reading. Finally, 12 articles with 1608 patients in total were included in the present meta-analysis based on the bias of the inclusion and exclusion criteria abovementioned [15–26]. The lack of sufficient data for analysis was the most common reason for exclusion during the full-text review process.

The characteristics of the included 12 studies are summarized in Table 1. Among the 12 eligible studies published from 2013 to 2020, one was from Ireland and the remaining 11 were conducted in China. Seven of 12 studies didn’t report the follow-up duration. Two study’s follow-up time were at least 40 months and 60 months respectively. The median follow-up duration is 54 months (range 1–122 months) in Seán Fitzgerald’s research, 60 months in Zhang Y’s study and 43.6± 38.4 months (range, 4–132 months) in Li CG’s work. Twelve studies reported the overall survival (OS) of patients with CRC, whereas two studies reported an association between recurrence-free survival (RFS) and TRIM proteins. The expression of TRIM proteins in tissue samples and adjacent non-tumor tissues was detected by immunohistochemistry. Most of the enrolled studies performed univariate and multivariate Cox regression analyses; otherwise, Kaplan–Meier Curves were provided. The crude and adjusted HR values reported by univariate and multivariate analysis and HR extracted by Kaplan-Meier curves are listed in Supplementary Table 1.

**Table 1:** Characteristics of included studies

**Author**	**Year**	**Country**	**Sample size**	**Over-** **expression**	**Detection method**	**Evaluation standard of TRIM overexpression**	**End-points** **(analysis type)**	**Follow-up**	**NOS score**
Seán Fitzgerald	2013	Ireland	137	42	IHC	staining intensity > 2 units of difference	OS, RFS	median follow up of 54 months (range 1–122 months)	6
Hong XW	2016	China	90	45	qPCR	median value of mRNA levels	OS	NR	8
Wang FQ	2017	China	97	61	IHC	percentage of positive cells > 25%	OS	NR	8
Ma SZ	2017	China	145	97	IHC	percentage of positive cells > 50%	OS	≥ 60 months	8
Zhang Y	2018	China	50	42	IHC	Final staining score > 4	OS	Median follow up of 60months	8
Wang HY	2018	China	87	44	IHC	Final staining score > 5	OS	NR	8
Chen DC	2018	China	374	314	IHC	Final staining score > 4	OS	NR	7
Li CG	2019	China	120	65	IHC	Final staining score > 1	OS	mean follow-up of 43.6± 38.4 months (range, 4–132 months).	8
Liang Q	2019	China	180	90	IHC	Final staining score > 6	OS	NR	8
Ding Y	2020	China	129	75	IHC	Final staining score > 6	OS, RFS	≥ 40 months	8
Han YD	2020	China	119	69	IHC	Final staining score > 3	OS	NR	8
Zhang SE	2020	China	90	56	IHC	percentage of positive cells > 25%	OS	NR	8

*IHC* Immunohistochemistry, *qPCR* Quantitative Real-time Polymerase Chain Reaction, *NR* Not reported

All enrolled articles, ranging from six to nine, were assessed to be of high quality based on the Newcastle–Ottawa quality assessment scale.

### Correlation between increased TRIM expression and OS

**Fig. 2:**
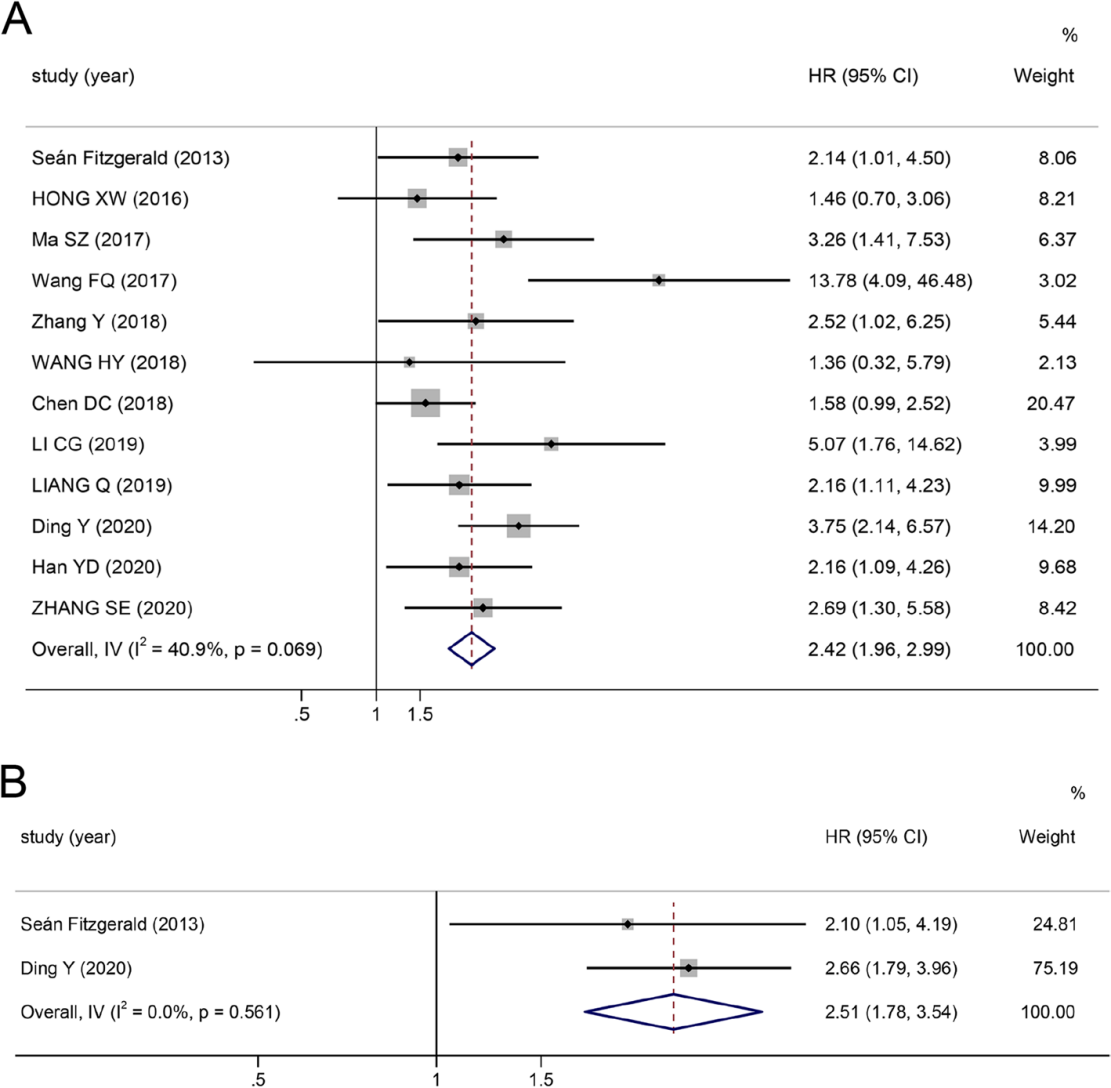
Forest plot of HR for the relationship between increased TRIM family expressions and OS and RFS

As is shown in Fig. 2, the summary HR for the OS comparing TRIM proteins over-expression versus low-expression is 2.42 (95% CI: 1.96–2.99, ***P ≤ 0.0001), and moderate heterogeneity between studies (I^2^ = 40.9%, *P* = 0.069) is observed. Elevated TRIM expressions in CRC tissues are strongly associated with poor OS, indicating that elevated TRIM protein levels could serve as a poor prognostic indicator in CRC. In addition, considering the presence of heterogeneity, subgroup analyses were performed according to the following three parameters: cut-off value (final staining scores, percentage of positive cells and others), sample size of studies (≥ 100 and < 100) and analysis type (multivariate and survival curves). Pooled analysis results demonstrated that all subgroups were in accordance with the OS as abovementioned (Supplementary Table 2); however, a possible interaction was noted between the analysis type and sample size subgroups. The heterogeneity was clearly decreased in the analysis type (I^2^ = 0.0%) and sample size (I^2^ = 27.1%) subgroups, suggesting that different types of analyses and sample capacity may result in the main heterogeneity among the correlations between the overexpression and OS.

### Correlation between increased TRIM expression and RFS

As shown in Fig. 2, the pooled HR demonstrates that a high level of TRIM expression is associated with poor RFS (HR 2.51, 95% CI: 1.78–3.54, ***P ≤ 0.0001). No heterogeneity was observed among the studies (I^2^ = 0.0%, *P* = 0.561).

### Correlation between increased TRIM expression and clinicopathological features

To assess the risk of TRIM overexpression under different clinicopathological features, the pooled odds ratios (ORs) were calculated (Table 2). The results revealed that a high-level expression of TRIM was associated with an advanced TNM stage (OR = 2.26, 95% CI: 1.25–4.10), deeper depth of tumor invasion (OR = 2.01, 95% CI: 1.04–3.88), poor tumor differentiation (OR = 0.63, 95% CI: 0.44–0.90), lymph node metastasis (OR = 2.99, 95% CI: 2.19–4.09) and perineural invasion (OR = 1.95, 95% CI: 1.18–3.23). Conversely, no significant association was observed between up-regulated TRIM expressions and gender (OR = 1.04, 95% CI: 0.82–1.33, *P* = 0.725), tumor location (OR = 0.93, 95% CI: 0.65–1.33, *P* = 0.673) or distant metastasis (OR = 0.92, 95% CI: 0.39–2.19, *P* = 0.855).

**Table 2:** Meta-analyses of up-regulated TRIM expressions and clinicopathological parameters

**Clinicopathological parameter**	**Studies (n)**	**OR (95% CI)**	*P***-value**	**Heterogeneity**		
				**I**^**2**^** (%)**	**P**_**Q**_	**Model**
Gender (male vs. female)	10	1.04 (0.82-1.33)	0.725	0.0	0.642	fixed
Tumor depth (T3-4 vs. T1-2)	7	2.01 (1.04-3.88)	0.039	71.2	0.002	random
Lymph node metastasis (+ vs. -)	7	2.99 (2.19-4.09)	<0.0001	0.0	0.798	fixed
Distant metastasis (+ vs. -)	9	0.92 (0.39-2.19)	0.855	66.2	0.003	random
TNM stage (III-IV vs. I-II)	9	2.26 (1.25-4.10)	0.007	77.2	0.0005	random
Poorly/undifferentiated vs. Well/moderately	7	0.63 (0.44-0.90)	0.01	38.4	0.136	fixed
Location (colon vs. rectum)	5	0.93 (0.65-1.33)	0.673	0.0	0.416	fixed
Perineural invasion (+ vs. -)	3	1.95 (1.18-3.23)	0.009	0.0	0.442	fixed

### Publication bias and sensitivity analysis

**Fig. 3:**
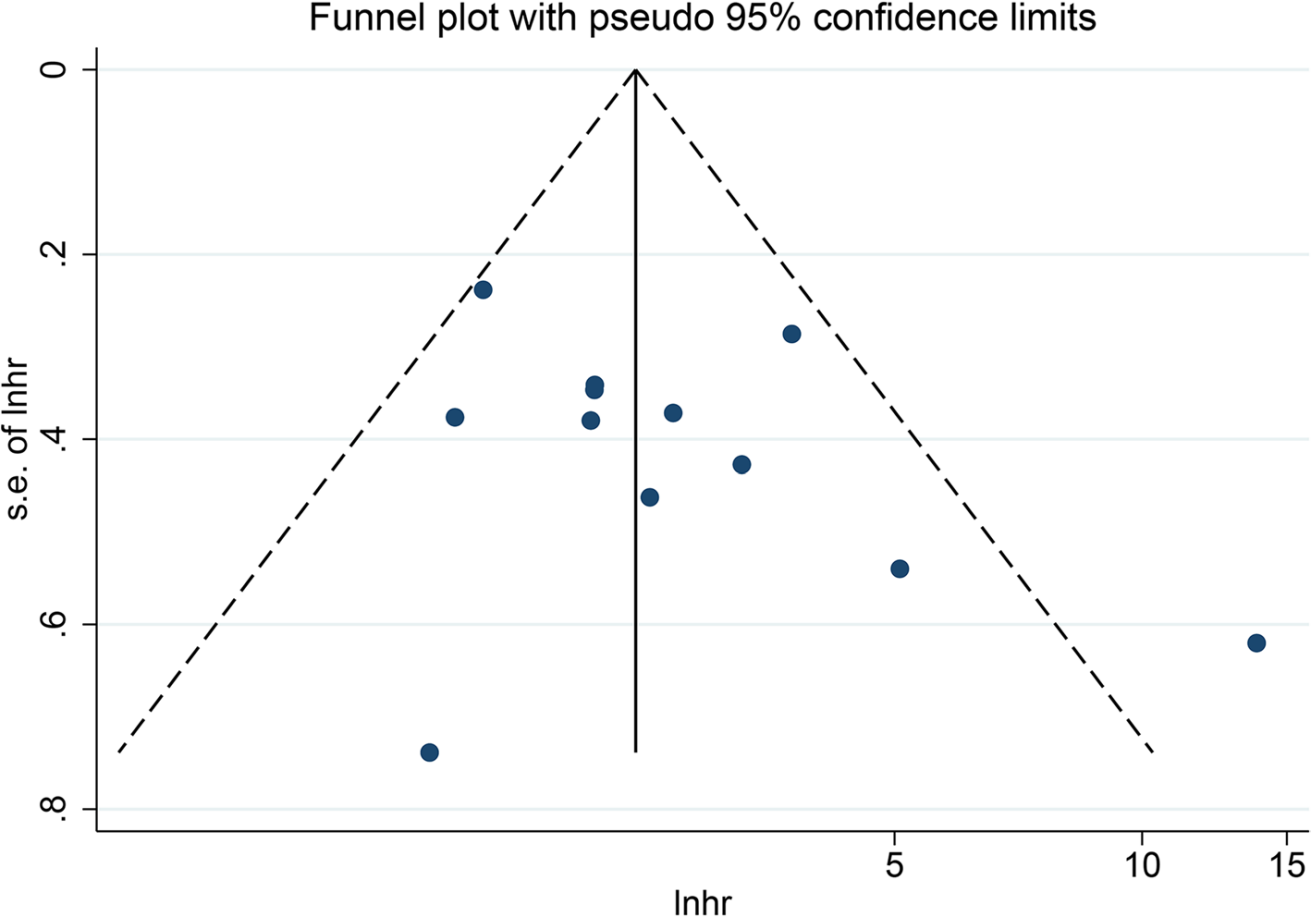
Funnel plot of TRIM family expressions and OS

**Fig. 4:**
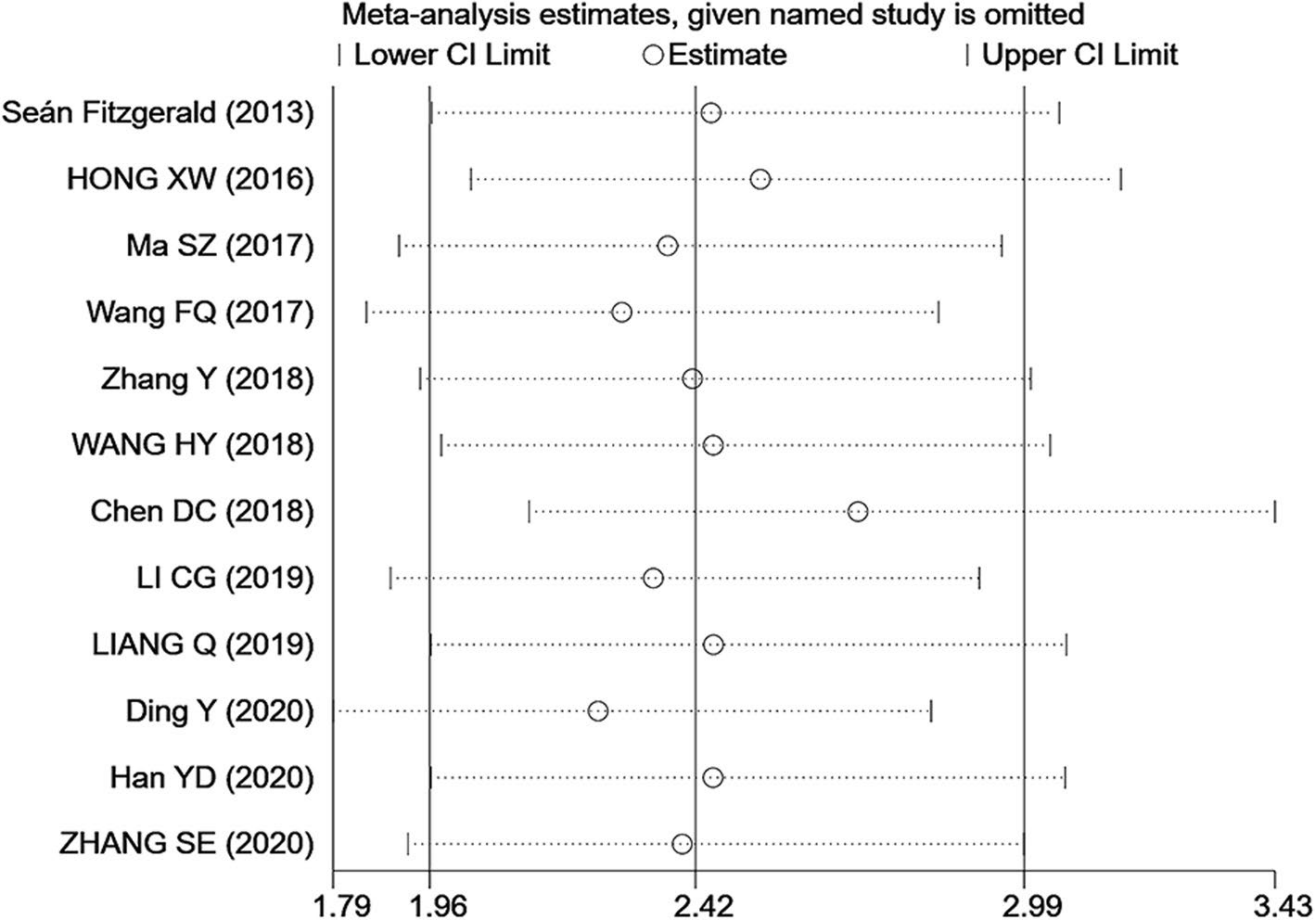
Sensitivity analysis of TRIM family expressions and OS

Funnel plot and Begg’s and Egger’s tests were used to test for publication bias. The funnel plot for OS did not show any obvious publication bias (Fig. 3). The results of Begg’s (*P* = 0.244) and Egger’s (*P* = 0.184) tests were not significantly different (Supplementary Figure S1); therefore, no significant publication bias occurred in the present meta-analysis. In addition, sensitivity analysis (Fig. 4) showed that even after sequentially omitting the included articles, the pooled results of the meta-analysis were still relatively robust.

## Discussion

Thus far, more than 80 TRIM proteins have been discovered and shown to be involved in the tumor progression of various malignancies [27]. Numerous TRIM proteins are implicated in the ubiquitination–proteasome system as E3-ubiquitin ligases. Therefore, they regulate diverse cellular processes, including proliferation, differentiation, signal transduction, autophagy and protein stability, which can result in tumorigenesis in an increasingly specific and important manner [8]. Numerous TRIM family members have been reported to be overexpressed in one or more cancers, such as hepatocellular carcinoma, gastric, lung and pancreatic cancers and breast carcinoma [28–30]. In contrast to non-tumor tissues, CRC tissues show higher levels of TRIM52 expression, and elevated TRIM52 levels are significantly correlated with the proliferation, migration and invasion of CRC cells [31]. TRIM59 is also upregulated and associated with tumor size and lymph node metastasis, indicating poor prognosis in CRC [32]. The upregulation of distinct TRIM family members is correlated with poor overall survival outcomes among cancer patients; therefore, TRIM proteins have excellent potential as biomarkers for cancer prognosis.

Recent studies have aggregated the functions of certain TRIM family members in cancer prognosis according to diverse tumor types. For instance, Hu *et al.* estimated the mortality risk in a relatively large population composed of 7239 participants in 17 studies, which showed that high expression of TRIM21 was significantly correlated with improved OS and progression-free survival in various cancers [33]. A meta-analysis by Xiao *et al.* reported that overexpression of TRIM44 was significantly correlated with not only shorter over-survival and worse disease-free survival, but also tumor metastasis, poor tumor differentiation, deeper tumor invasion and advanced clinical stage [34]. Both Xue *et al.* and Yuan *et al.* reported that upregulated TRIM24 expression significantly predicts poor overall survival in different types of malignancies [35, 36]. Whether the overexpression of TRIM proteins could be useful in predicting the prognosis of patients with CRC remains unclear.

To our knowledge, our meta-analysis is the first systematic review and meta-analysis to assess the prognostic and clinicopathological values of TRIM proteins for patients with CRC. Herein, the pooled results of 1608 participants with CRC from 12 eligible studies indicated that patients with CRC with elevated TRIM protein expressions had significantly shorter OS and worse RFS than those with normal TRIM expressions. In the meta-analysis of OS, moderate heterogeneity was observed (I^2^=40.9%, *P* = 0.069). However, subsequent subgroup analyses further confirmed that the cut-off value, sample size and type of analysis did not significantly affect the results. Moreover, sensitivity analyses were performed by omitting one study at a time, and publication bias tests did not significantly influence the results. Therefore, the overexpression of TRIM proteins may be positively correlated with poor prognosis. TRIM28, which is highly expressed in patients with CRC, is an independent prognostic marker for OS, with a pooled HR of 2.57 in two different prospective studies by Fitzgerald et al. and Ma et al., with a total of 202 patients with CRC (Supplementary Figure S2) [15, 17].

Because the clinicopathological features in different groups of patients affect their outcomes, eight major factors between patients with upregulated and normal TRIM expressions were compared. The overexpression of TRIM proteins was markedly associated with higher TNM stage, deeper invasion, lymph node metastasis and perineural invasion. Notably, patients with higher levels of TRIM proteins exhibited an enhanced degree of tumor histological differentiation, which contradicts previous studies [34]. Thus, more studies with larger populations should be conducted in the future to confirm these conflicting results. However, TRIM protein expression was not significantly associated with gender, tumor site or distant metastasis. In the present study, overexpression of TRIM proteins was associated with CRC progression and poor clinical outcomes. An increasing number of studies have demonstrated that TRIM proteins contribute to the diverse malignant biological behaviors of CRC cells. Hence, TRIM proteins have excellent potential to serve as biomarkers for CRC prognosis, and further research should be conducted to investigate the molecular mechanisms and to verify the clinical application of TRIM proteins.

Our systematic review has some limitations. First, most of the studies we chose were conducted in the Chinese population, to the extent that the statistical analysis of publication bias lacked sufficient power. Notably, one study with a high HR was responsible for almost all the observed heterogeneity. However, sensitivity analyses showed that removing this article did not significantly influence the pooled results. Therefore, additional samples from other countries or races should be included to confirm the reliability of these results. Second, we were unable to contact the authors of certain studies to retrieve data. In some articles, HRs and 95% CI were extracted from additional data, such as Kaplan–Meier curves, which might have resulted in an uncertain bias for the pooled estimates. And unfortunately, some studies lack of detailed information, which might result in a lack of control for potential confounding factors. Third, owing to the limited number of studies, heterogeneity still existed in some results of clinicopathological characteristics.

## Conclusion

In summary, our current study demonstrates that TRIM proteins are correlated with cancer progression and prognosis in patients with CRC. The TRIM family may be a reliable biomarker for determining the clinicopathological characteristics and prognosis of malignancies. With further understanding of the regulatory mechanisms, TRIM proteins are promising new therapeutic targets for CRC.

### Supplementary Information

**Supplementary material 1.**

## Data Availability

All data generated or analysed during this study are included in this published article and its Supplementary information files.
