# Supplementary material for: Association of the TRIM family protein with survival outcomes and clinicopathological features in colorectal cancer: a systematic review and meta-analysis
Source: BMC Cancer. 2024 Apr 27;24:537. doi: 10.1186/s12885-024-12280-z (PMC11055242; doi:10.1186/s12885-024-12280-z)
Supplement: Supplementary file 1 — Supplementary material 1. [file 12885_2024_12280_MOESM1_ESM.doc]

**Supplementary Fig. S1.** Begg’s test and Egger’s test for publication bias assessment of TRIM proteins and OS.


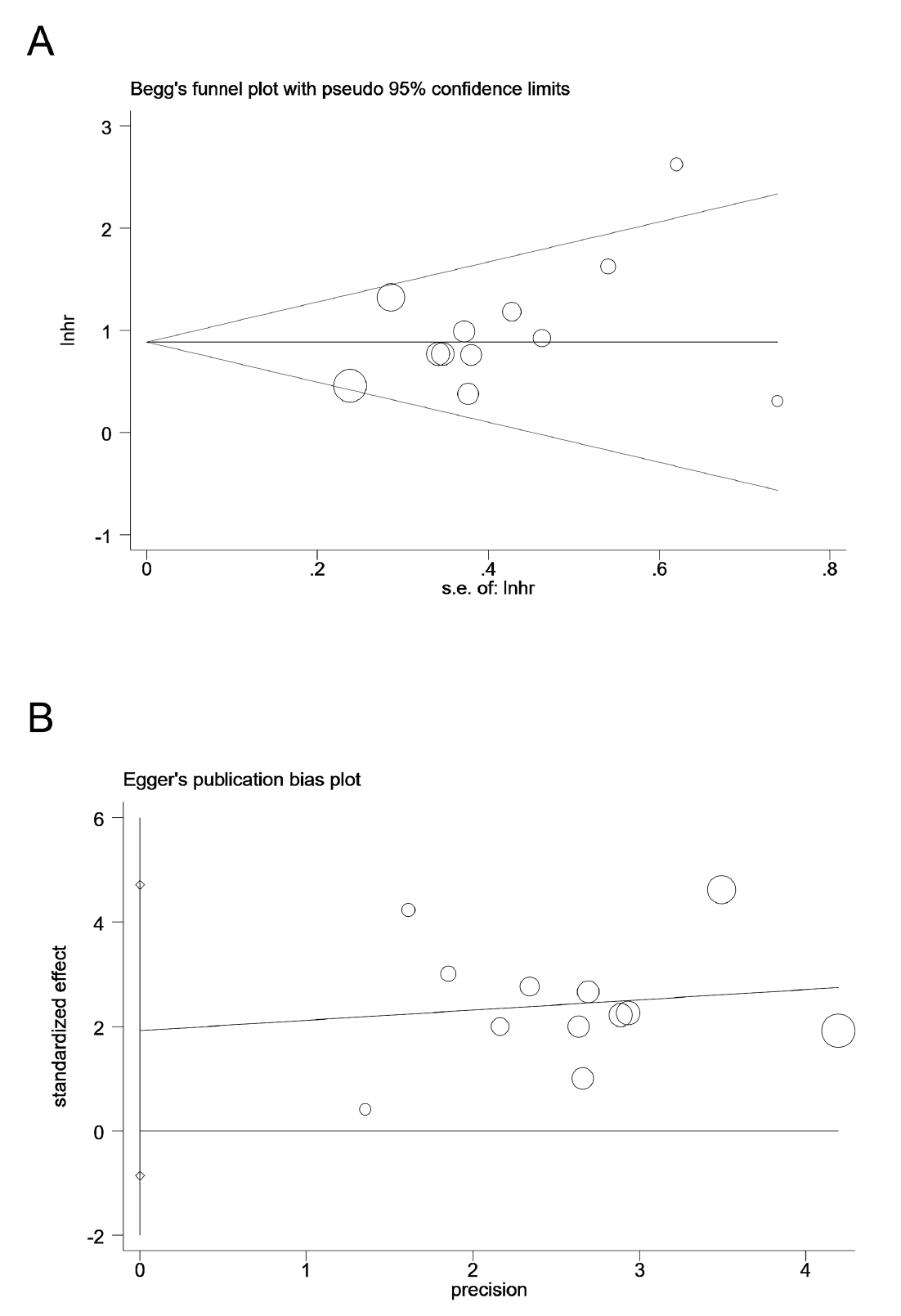


**Supplementary Fig. S2.** Forest plot of HR for the relationship between increased TRIM28 and OS.


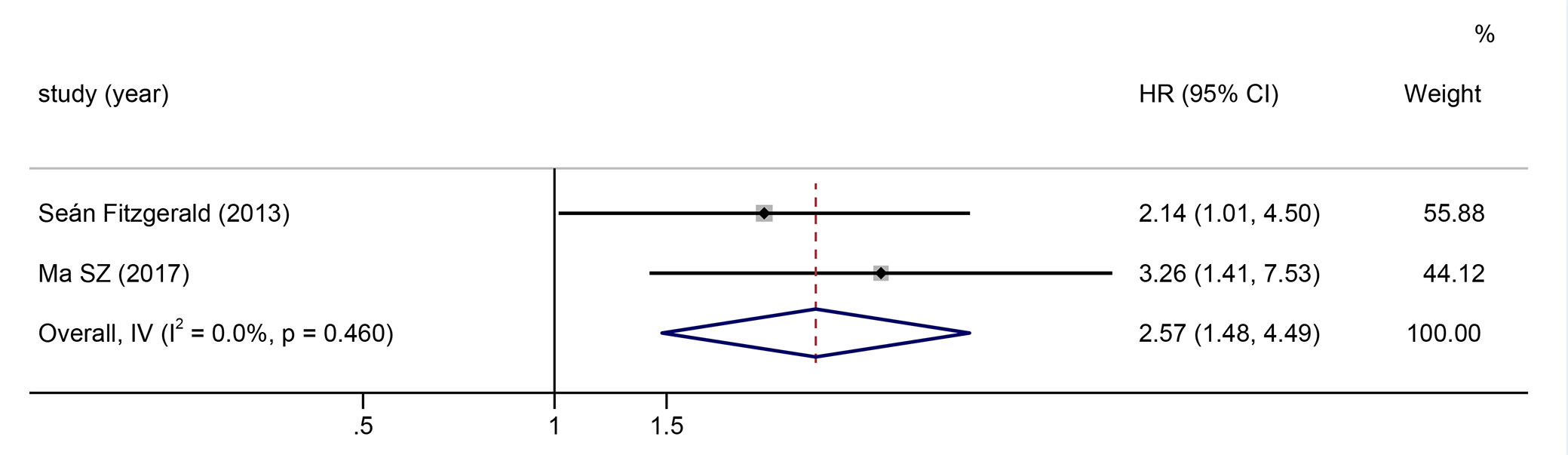


**Supplementary Table 1.** Univariate (crude HR) and multivariate (adjusted HR) analysis of OS and RFS.

| **Author** | **OS - univariate** | | **OS - multivariate** | | **RFS - univariate** | | **RFS -** **multivariate** | | **Adjusted factors** |
| --- | --- | --- | --- | --- | --- | --- | --- | --- | --- |
|  | **HR** | **95%CI** | **HR** | **95%CI** | **HR** | **95%CI** | **HR** | **95%CI** |  |
| Seán Fitzgerald | 2.070 | 1.036-4.137 | 2.136 | 1.015-4.498 | 1.944 | 1.005-3.759 | 2.100 | 1.052-4.191 | Gender, age, T-stage, N-stage，M-stage |
| Hong XW |  |  | 1.46 | 0.70-3.06 |  |  |  |  | NR |
| Wang FQ |  |  | 13.782 | 4.087-46.476 |  |  |  |  | Tumor size, clinical stage, CEA level |
| Ma SZ |  |  | 3.26 | 1.41-7.53 |  |  |  |  | Gender, age, differentiation degree, depth of invasion, Dukes stage, lymph nodes metastasis, distant metastasis |
| Zhang Y | 2.63 | 1.14-6.05 | 2.52 | 1.02-6.25 |  |  |  |  | Depth of invasion, lymph node metastasis, liver metastasis |
| Wang HY |  |  | 1.36 | 0.32-5.79 |  |  |  |  | NR |
| Chen DC | 2.34 | 1.18-4.63 | 1.58 | 0.99-2.2 |  |  |  |  | Gender, age, differentiated degree, clinical stage，relapse and metastasis, pT status, pN status, pM status |
| Li CG | 7.710 | 2.709-21.947 | 5.075 | 1.762-14.623 |  |  |  |  | T stage, N stage, M stage, lymphovascular invasion |
| Liang Q | 2.41 | 1.25-4.66 | 2.16 | 1.11-4.23 |  |  |  |  | Distant metastasis, lymph node metastasis, histological differentiation, AJCC stage |
| Ding Y | 3.96 | 2.28-6.90 | 3.75 | 2.14-6.57 | 2.78 | 1.88-4.13 | 2.66 | 1.79-3.96 | Differentiated degree, depth of invasion, lymph node metastasis, TNM stage, lymphatic vessel invasion, perineural invasion |
| Han YD | 2.514 | 1.324-4.773 | 2.158 | 1.094-4.257 |  |  |  |  | AJCC stage |
| Zhang SE |  |  | 2.69 | 1.30-5.58 |  |  |  |  | CEA levels, clinical stage, tumor size |

NR: not reported.

**Supplementary Table 2.** Subgroup analyses of the association between TRIM expressions and OS.

| **Categories** | **Studies (n)** | **Number of**  **patients** | **HR (95% CI)** | **P-value** | **Heterogeneity** | | |
| --- | --- | --- | --- | --- | --- | --- | --- |
|  |  |  |  |  | **I^2^ (%)** | **P_Q_** | **Model** |
| All | 12 | 1608 | 2.42 (1.96-2.99) | <0.0001 | 40.9 | 0.069 | fixed |
| Cut-off for over expression |  |  |  |  |  |  |  |
| Final staining scores | 3 | 275 | 2.81 (1.76-4.49) | <0.0001 | 0.0 | 0.909 | fixed |
| Percentage of positive cell | 7 | 1106 | 2.51 (1.92-3.27) | <0.0001 | 62.6 | 0.013 | fixed |
| Others | 2 | 227 | 1.76 (1.04-2.98) | 0.034 | 0.0 | 0.477 | fixed |
| Analysis type |  |  |  |  |  |  |  |
| Multivariate | 10 | 1342 | 2.90 (2.26-3.73) | <0.0001 | 22.3 | 0.238 | fixed |
| Survival curves | 2 | 266 | 1.54 (1.04-2.29) | 0.031 | 0.0 | 0.859 | fixed |
| Sample size |  |  |  |  |  |  |  |
| >100 | 7 | 1204 | 2.39 (1.87-3.06) | <0.0001 | 27.1 | 0.222 | fixed |
| ≤100 | 5 | 404 | 2.51 (1.67-3.76) | <0.0001 | 61.3 | 0.035 | fixed |
